# Is post-trabeculectomy hypotony a risk factor for subsequent failure? A case control study

**DOI:** 10.1186/1471-2415-5-7

**Published:** 2005-04-05

**Authors:** Sarah E Benson, Kaveri Mandal, Catey V Bunce, Scott G Fraser

**Affiliations:** 1 Sunderland Eye Infirmary, Queen Alexandra Road, Sunderland, SR2 9HP, UK; 2 Moorfields Eye Hospital, City Road, London, EC1V 2PD, UK; 3 School of Health, Natural and Social Sciences, University of Sunderland, UK

## Abstract

**Background:**

Ocular hypotony results in an increased break down of the blood-aqueous barrier and an increase in inflammatory mediator release. We postulate that this release may lead to an increased risk of trabeculectomy failure through increased bleb scarring. This study was designed to try to address the question if hypotony within one month of trabeculectomy for Primary Open Angle Glaucoma (POAG), is a risk factor for future failure of the filter.

**Methods:**

We performed a retrospective, case notes review, of patients who underwent trabeculectomy for POAG between Jan 1995 and Jan 1996 at our hospital. We identified those with postoperative hypotony within 1 month of surgery. Hypotony was defined as an intraocular pressure (IOP) < 8 mmHg or an IOP of less than 10 mmHg with choroidal detachment or a shallow anterior chamber. We compared the survival times of the surgery in this group with a control group (who did not suffer hypotony as described above), over a 5 year period. Failure of trabeculectomy was defined as IOP > 21 mmHg, or commencement of topical antihypertensives or repeat surgery.

**Results:**

97 cases matched our inclusion criteria, of these 38 (39%) experienced hypotony within 1 month of surgery. We compared the survival times in those patients who developed hypotony with those who did not using the log-rank test. This data provided evidence of a difference (P = 0.0492) with patients in the hypotony group failing more rapidly than the control group.

**Conclusion:**

Early post-trabeculectomy hypotony (within 1 month) is associated with reduced survival time of blebs.

## Background

Glaucoma is one of the leading causes of blindness in the world [1,2]. Medical therapies are the mainstay of treatment for primary open angle glaucoma, however resistant cases may require surgical intervention [3]. Trabeculectomy has been the preferred surgical procedure for uncontrolled glaucoma since its introduction in 1967 by Cairns.

Although the procedure has undergone various modifications, the principle behind trabeculectomy remains the same. It is a filtering procedure that provides a fistula for drainage of aqueous humour via the sub-conjunctival space, into the conjunctiva and subsequent absorption by the surrounding tissues [4]. Ocular hypotony due to over-filtration is a well documented and sight-threatening complication of trabeculectomy [5,6].

To our knowledge there has been no publications looking at the long-term outcome of eyes that have had trabeculectomy with early (less than one month) hypotony. We hypothesise that hypotony in this early postoperative period could result in an increased breakdown of the blood aqueous barrier compared to non- hypotonous post trabeculectomy eyes. This could allow release of inflammatory mediators into the aqueous and through the filtering bleb into the surrounding tissues. These mediators could cause scarring thereby resulting in failure of the trabeculectomy. The purpose of our investigation was to compare the survival times of trabeculectomy in two groups of patients, those who experienced hypotony in the early postoperative period and those who did not.

## Methods

The notes of patients undergoing trabeculectomy for primary open angle glaucoma (POAG) from Jan 95 to Jan 96 at Sunderland Eye Infirmary were reviewed. All surgeons (8) were Consultant grade, and none (at the time of the study) had a special interest in glaucoma.

Exclusion criteria included normal pressure glaucoma, secondary glaucomas, combined phacotrabeculectomies, and augmented surgery (with Mitomycin-C and 5-Fluorouracil). All cases with pre-operative shallow anterior chambers were also excluded, although anterior chamber depth was not formally measured. To minimise confounding factors and make as homogenous a study group as possible we also excluded previous intraocular surgery, any previous intraocular inflammation and prior argon laser trabeculoplasty.

For the purpose of our study, hypotony was defined as an IOP of < 8 mmHg or an IOP < 10 mmHg with choroidal detachment or shallow anterior chamber (with either iris-cornea touch, or lens-cornea touch).

### Two distinct groups of patients were identified

1) Control group were those who did not experience hypotony within a month of their operation or at any stage postoperatively.

2) Study group included patients who had IOP < 8 mmHg alone or an IOP < 10 mmHg with choroidal detachment or a shallow anterior chamber within one month after trabeculectomy.

We defined failure of trabeculectomy as IOP > 21 mmHg which was persistent (recorded at more than 3 occasions) at or after 6 months postoperatively, or commencement of topical antihypertensive agents, or repeat surgery.

## Results

97 cases matched the inclusion criteria. These patients were from the population of Sunderland and its surrounding areas. The cases in both the control and hypotonous groups were exclusively Caucasian and there was no evidence of difference in mean age, gender or median IOP between the two groups. 39% (38/97) cases experienced hypotony within the 1^st ^month postoperative whilst 61% (59/97) did not.

**Figure 1:**
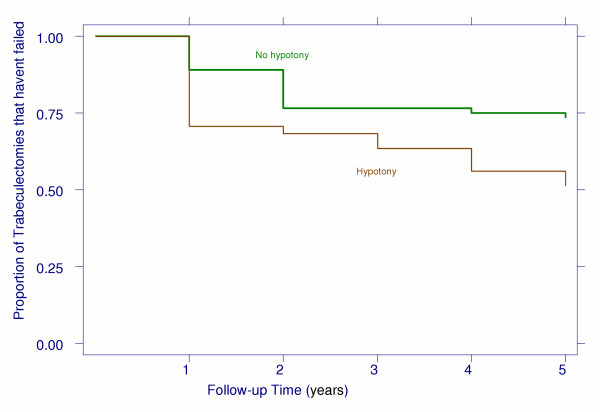
Kaplan Meier 5 year Survival of Trabeculectomies.

The notes of patients in both groups were reviewed for each visit until 5 years postoperatively. The cases that failed and the duration of survival of their filters were noted for the control and study group. We compared the survival times in those patients who developed hypotony with those who did not using the log-rank test. A Kaplan Meier curve illustrates their 5 year survival (Fig 1).

This data provided evidence of a statistically significant difference (P = 0.0492) with patients in the hypotony group failing more rapidly than the control group.

**Figure 2:**
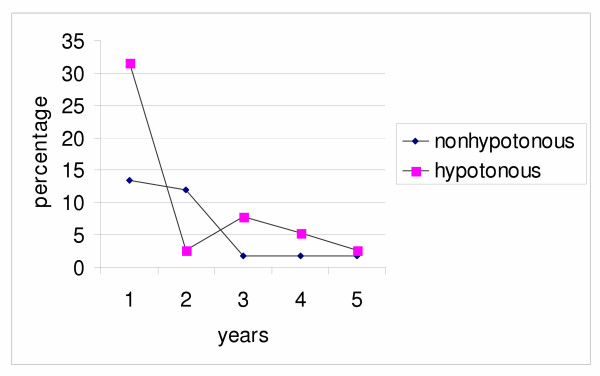
Percentage Of New Failures Of Trabeculectomies Each Year Post surgery.

Figure 2 indicates the number of trabeculectomies that failed in each year of the study and it can be seen that most failed within the first year – especially in the hypotonous group.

## Discussion

Postoperative hypotony, flat anterior chamber and choroidal haemorrhage are known complications of full thickness filtration procedures [7-10]. Trabeculectomy was introduced to limit these. Scarring of the filtering bleb by fibrous tissue is the most common cause of long term failure in glaucoma filtration surgery [11]. This is commoner in younger patients [12] and in African Caribbean races [13,14].

Use of adjunctive antimitotic agents such as 5-FU and Mitomicin C maintain the filtration bleb by preventing fibrosis thereby achieving a lower intraocular pressure compared to those trabeculectomies performed without the use of these agents. They frequently cause transient hypotony in the initial 4–6 weeks [15-17] and were excluded from our study.

Eyes with previous uveitis or multiple intraocular surgeries can develop hypotony secondary to growth of a cyclitic membrane in the ciliary body[18]. Inflammation in the anterior chamber angle could precipitate ciliary body shutdown thereby resulting in reduced aqueous production and ultimately hypotony in these cases. Trabeculectomy in uveitic eyes are also at a higher risk of failure[19]. In order to try to reduce the variables in our investigation, eyes with previous uveitis and intraocular surgery were excluded.

Early post-trabeculectomy hypotony can result from various factors such as over-filtration caused by loose closure of the scleral flap, conjunctival bleb leak, ciliochoroidal detachment [16,17], hyposecretion of aqueous humour accompanying ocular inflammation [15] and cyclodialysis cleft [18,20]. Trabeculectomy can result in ciliary body shutdown [21] that leads to intraocular inflammation and breakdown of the blood aqueous barrier thereby resulting in hypotony in the early postoperative period.^18 ^Rarer causes of early postoperative hypotony include retinal detachment and globe perforation[18].

Post-trabeculectomy hypotony has been associated with many complications such as accelerated cataract progression [18,22], ciliochoroidal effusion, choroidal haemorrhage, corneal changes [19], inflammation, prolonged recovery of visual acuity [5] and hypotony maculopathy with marked visual loss[18]. Dellaporta [23] and later Gass [24] described the retinal manifestations of hypotony such as optic disc swelling, chorioretinal folds and changes in retinal pigment epithelium. Gass described these findings as 'hypotony chorioretinopathy'. Our series also suggests, as a further complication of post-trabeculectomy hypotony, reduced survival time of non-augmented filtering blebs.

**Figure 3:**
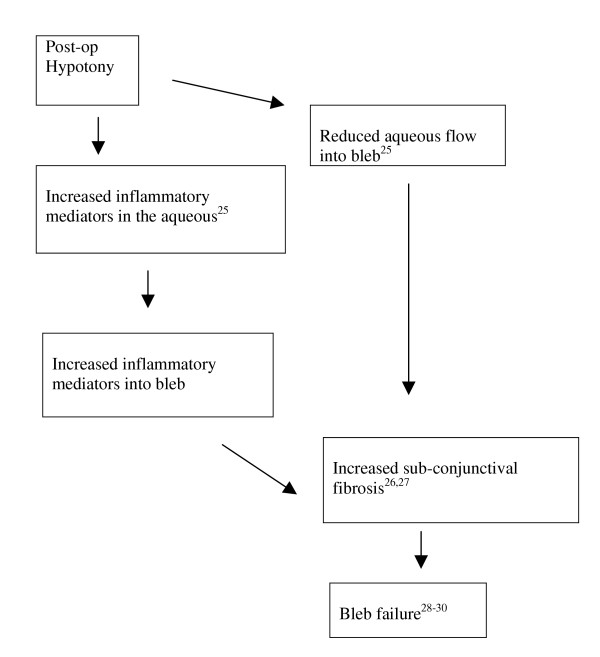
Flow diagram of the suggested pathogenesis of bleb failure after hypotony.

Hypotony is associated with an alteration in aqueous flow and its composition – partly because it initiates an inflammatory response[25]. This means that in the hypotonous eye not only will there be a reduced flow of aqueous through the sclerostomy and into the bleb, but the aqueous that does pass into the bleb will have even more inflammatory mediators than normally occurs post-operatively. This reduction in flow and increase in inflammatory mediators are likely to be potent causes of sub-conjunctival scarring and subsequent failure[26]. This is the mechanism that we propose for our finding that post-trabeculectomy hypotony results in increased chance of failure of the filter and is summarised in figure 3.

In our investigation, a statistically significantly higher proportion of trabeculectomies experiencing postoperative hypotony failed within 5 years compared to the control group. These findings have implications for surgical practice such as tight suturing of scleral flaps to limit egress of aqueous into the bleb and meticulous suturing of the overlying conjunctiva to prevent bleb leakage.

As far as literature is concerned Schwenn et al [5] conducted a prospective 1 year follow-up of 43 eyes undergoing trabeculectomy. Contrary to our findings, their results suggested that early hypotony was more likely to result in better IOP control. However, our population base was larger and follow-up period longer.

Stewart and Shields [27] followed 36 eyes that had undergone trabeculectomy, they studied the postoperative anterior chamber depth and complication rate. They concluded that a flat anterior chamber with cornea-lens touch resulted in a significantly higher rate of complications, including corneal oedema, cataract, and bleb failure. Those eyes with iris-cornea touch were allowed to reform their anterior chambers spontaneously, and had a similar outcome to eyes that maintained an anterior chamber throughout the follow up. These findings may seem contradictory to our results, however our definition of hypotony does not rely on anterior chamber depth alone. To satisfy this group an eye in our series required an IOP of less than 10 mmHg along with a shallow anterior chamber or choroidal detachment or IOP less than 8 mmHg. Our patient group may have had more severe post-operative hypotony.

Stewart and Shields patient group had a diverse range of glaucoma diagnoses as opposed to our homogenous group of primary open angle glaucoma, therefore direct comparisons are inappropriate. In addition their follow up was for 3 months only and their sample size only 36.

A randomised controlled trial, usually the best way to reduce potential biases, would be ethically inappropriate in this clinical situation.

The limitations of our study include consecutive patients who underwent trabeculectomy for POAG in the one year period could not be included as 24 were lost to follow-up, 2 developed endophthalmitis and 2 required vitreoretinal surgery. Those 24 patients for whom we had incomplete data were fairly evenly distributed for the hypotonous (n = 10) and the non-hypotonous (n = 14) groups.

We did not collect data on the pre-operative topical drug regime. The use of two or more antiglaucoma medications is a risk factor for failure of filtration surgery [19]. Broadway et al have shown that not only does the number of prior topical antiglaucoma medications, but also the duration of use of these agents affect the success of trabeculectomy [28-31].

## Conclusion

Hypotony results in the break down of blood-aqueous barrier causing the release of inflammatory mediators. Persistent conjunctival inflammation induces enhanced bleb scarring [26,32]. Excessive conjunctival scarring significantly reduces the success of surgery [33-35].

In our series, early post-trabeculectomy hypotony (within 1 month) is associated with reduced bleb survival time over 5 years. Most of the cases of failure in the hypotonous group occurred in the first year postoperatively.

This study is further evidence that hypotony is detrimental to the eye. Although further investigation is required, it may be that even though hypotony resolves in the short term, there is a risk of longer term filter failure. Avoidance of hypotony with measures such as meticulous closure of the scleral flap (but still allowing aqueous flow) and of the conjunctiva remain essential steps for short and long term bleb functioning.

## Competing interests

The author(s) declare that they have no competing interests.

## Authors' contributions

Data was collected by Sarah Benson and Kaveri Mandal. Statistical analysis was performed by Catey Bunce. Scott Fraser conceived of the study, and participated in its design and coordination and helped to draft the manuscript, along with Sarah Benson and Kaveri Mandal. All authors read and approved the final manuscript.

## Pre-publication history

The pre-publication history for this paper can be accessed here:
